# Researcher perspectives on challenges and opportunities in conservation physiology revealed from an online survey

**DOI:** 10.1093/conphys/coab030

**Published:** 2021-04-28

**Authors:** Christine L Madliger, Oliver P Love, Vivian M Nguyen, Neal R Haddaway, Steven J Cooke

**Affiliations:** 1 Department of Biology and Institute of Environmental and Interdisciplinary Science, Carleton University, 1125 Colonel By Dr., Ottawa, Ontario, K1S 5B6, Canada; 2 Department of Integrative Biology, University of Windsor, 401 Sunset Ave., Ontario, N9B 3P4, Canada; 3 Stockholm Environment Institute, Linnégatan 87D, 10451 Stockholm, Sweden; 4 Mercator Research Institute on Global Commons and Climate Change, Torgauer Strasse 19, 10829, Berlin, Germany; 5 Africa Centre for Evidence, University of Johannesburg, Johannesburg, 2092, South Africa

**Keywords:** Barrier, challenge, conservation physiology, conservation science, opportunities, success

## Abstract

Conservation physiology represents a recently emerging arm of conservation science that applies physiological tools and techniques to understand and solve conservation issues. While a multi-disciplinary toolbox can only help to address the global biodiversity crisis, any field can face challenges while becoming established, particularly highly applied disciplines that require multi-stakeholder involvement. Gaining first-hand knowledge of the challenges that conservation physiologists are facing can help characterize the current state of the field and build a better foundation for determining how it can grow. Through an online survey of 468 scientists working at the intersection of physiology and conservation, we aimed to identify characteristics of those engaging in conservation physiology research (e.g. demographics, primary taxa of study), gauge conservation physiology’s role in contributing to on-the-ground conservation action, identify the perceived barriers to achieving success and determine how difficult any identified barriers are to overcome. Despite all participants having experience combining physiology and conservation, only one-third considered themselves to be ‘conservation physiologists’. Moreover, there was a general perception that conservation physiology does not yet regularly lead to tangible conservation success. Respondents identified the recent conceptualization of the field and the broader issue of adequately translating science into management action as the primary reasons for these deficits. Other significant barriers that respondents have faced when integrating physiology and conservation science included a lack of funding, logistical constraints (e.g. sample sizes, obtaining permits) and a lack of physiological baseline data (i.e. reference ranges of a physiological metric’s ‘normal’ or pre-environmental change levels). We identified 12 actions based on suggestions of survey participants that we anticipate will help deconstruct the barriers and continue to develop a narrative of physiology that is relevant to conservation science, policy and practice.

## Teaser

We used an online survey of researchers to determine the challenges that the field of conservation physiology currently faces. We found that many participants cited communication difficulties, lack of funding, logistical constraints and a lack of physiological baseline data as barriers. We provide recommendations for overcoming some of these challenges.

## Introduction

Conservation science inherently involves combining various disciplines (e.g. conservation genetics, conservation behaviour, conservation social science; see Kareiva and Marvier, 2012) to solve complex problems, which is both laudable and necessary (Lubchenco, 1998; Dick *et al*., 2016). The field of conservation physiology seeks to apply physiological tools, techniques and knowledge to identify and solve conservation challenges (Cooke *et al*., 2013). It is relatively new, having only been named and described as a discipline with cohesive goals ~15 years ago (Wikelski and Cooke, 2006). It is clear that the field is growing: it now boasts a dedicated journal and textbook (Madliger *et al*., 2021), is increasingly represented at international scientific conferences and includes many early-career researchers identifying conservation physiology as the main focus of their research programmes. Nonetheless, given the nascent nature of the field, the fact that it merges two often-disparate sub-disciplines, and its mission-oriented goal of contributing to on-the-ground conservation action, it could inherently face a number of challenges (Cooke and O’Connor, 2010).

For the similarly interdisciplinary field of conservation behaviour, Caro and Sherman (2013) outlined 18 reasons why animal behaviourists may avoid working in the realm of conservation science. These barriers included a lack of targeted funding, differences in scale of study (e.g. individuals versus populations), lack of expertise and a perception that conservation science is less intellectually stimulating. We anticipate that physiologists may cite similar reasons as to why incorporating conservation applications as a component of their research goals is challenging and that conservation scientists may be hesitant to employ physiological techniques due to a lack of baseline physiological data (i.e. reference ranges of a physiological metric’s ‘normal’ or pre-environmental change levels) and the invasive nature of some physiological techniques (Cooke and O’Connor, 2010; Lennox and Cooke, 2014; Madliger and Love, 2015). With the conservation physiology toolbox rapidly expanding in terms of the number of tools available and their validation (Madliger *et al*., 2018), it is a worthwhile time to ascertain the challenges in the discipline that could be hindering growth to ensure that this toolkit can be applied as extensively as possible to promote conservation gains.

We are unaware of any attempt to survey scientists across the globe about their experiences navigating the field of conservation physiology, despite there being immense potential to gain information that is not readily shared in publications, focus groups or other forums. Identifying the specific challenges that researchers are facing in the field could indicate where misconceptions lie, provide information on which validations need to be performed to better apply physiology to conservation endeavours and provide starting points for improvement in communication (Cooke *et al.*, 2020). Explicitly articulating barriers can also represent a way to share frustrations; knowing others are experiencing similar challenges can strengthen the feeling of community among conservation physiologists and provide a rallying point to share strategies and approaches (McMillan and Chavis, 1986; Michaut, 2011). Overall, identifying barriers and opportunities allows researchers and practitioners to prioritize challenges and view them in a more objective way for problem-solving purposes, further helping to create a community of practice (McMillan and Chavis, 1986). Additionally, exploring the perspectives of those working across an entire discipline (i.e. physiology) rather than simply with a specific research tool (i.e. biotelemetry; as per Young *et al*., 2018) has the potential to identify what researchers could do to make meaningful advances in conservation practice and policy.

To begin better characterizing the field of conservation physiology and identifying where challenges exist, we surveyed scientists (using an online survey) with experience at the intersection of physiology and conservation science to identify the following: (i) the extent to which researchers engage in conservation physiology work and their demographic composition; (ii) the barriers researchers have experienced when integrating physiology and conservation science and the level of difficulty they have faced in overcoming them; (iii) whether participants believe conservation physiology is accomplishing its primary goals; and (iv) whether their own work linking physiology and conservation has led to on-the-ground conservation success. We conclude with recommendations for addressing the challenges the survey unveiled that also arose from ideas shared by survey respondents.

We recognize that there would also be value in conducting a similar survey with conservation practitioners, but that was beyond the scope of the current study. In addition, we note that a future study that enables hypothesis testing using quantitative tools would be desirable; however, this introductory survey to highlight possible barriers and opportunities was not designed to do so. Instead, we consider this to be a relatively modest, exploratory study that can be used to identify hypotheses worthy of formal testing in a more extensive follow-up endeavour. As such, we consider this a ‘perspective’ article in that we are synthesizing and sharing the perspectives of the members of the conservation physiology research community.

## Materials and Methods

### Participant pool

To form a pool of potential participants with experience working in the realm of conservation physiology, we performed a search in Web of Science (Core Collection) to identify research articles that combined physiology and conservation tools and approaches. We completed 4 separate searches on 1 December 2016 with the goal of identifying papers published in the following: (i) conservation journals that used physiological approaches; (ii) physiology journals that considered conservation implications; (iii) general ecology journals that combined physiological and conservation science approaches; and (iv) any scientific journal that used the term ‘conservation physiology’. The search strings we used for each scenario can be viewed in the Supplementary Information (Part 1). We retained all 3287 results from search (i), the first 3000 results from search (ii) (sorted on relevance), 3000 results from search (iii) and all 134 results from search (iv). We chose the number of results to retain to balance the contribution of papers across each search type and by examining the search results to choose a cut-off when results were no longer relevant (i.e. results were not fulfilling the above search criteria). Through the metadata stored in the Web of Science results, we extracted the email addresses of the corresponding authors on all 9421 publications, published between 1997 and 2016. After deleting duplicate emails, we reached a final potential participant email list of 7080.

### Survey instrument

We conducted an anonymous, international online survey (Supplementary Information, Part 2) of scientists, which was approved by the University of Windsor’s Research Ethics Board (#16-193) with adjunct clearance from the Carleton University Research Ethics Board-B (CUREB-B). The findings we present here are reported in aggregate, although we use quotes from open-ended questions to provide context. No self-identifying information was collected from participants. The survey was available from 5 December 2016 to 17 January 2017 and was administered via FluidSurveys. Participants were sent an email on 5 December 2016 inviting them to participate in the survey, with reminders sent on 19 December 2016 and 9 January 2017.

Of 707 individuals that opened the survey, 180 were filtered outside of the desired sample due to a response of ‘no’ to an initial question: ‘*Have you ever participated in research or other work that combines physiology and conservation?*’ Of the remaining participants, 468 completed the survey and submitted their responses. As a result, the overall response rate was 9%, which is similar to other targeted e-mail-based surveys (e.g. Cooke *et al*., 2016; Sappleton and Lourenco, 2016), which notoriously have lower response rates than mail surveys (Coderre *et al*., 2004). We cannot exclude the possibility that some spam filters categorized our survey invitations as ‘junk mail’, or that there was survey fatigue within the scientific community. We did not track the country of origin or any demographic parameters for individuals on our initial recruitment list, so it is not possible to determine if there was any geographic or demographic bias in the respondents. As stated above, participants were drawn from a pool of research publications that spanned 1997–2016, and we acknowledge that the period when researchers worked at the interface between physiology and conservation could influence their conceptualization of the field; however, we are unable to ascertain whether this bias existed in our data. The survey was only administered in English so we must assume that it is biassed towards researchers with a command of English. In addition, because we generated our survey panel by using published authors, our sample is inherently biassed towards those scientists who publish their work in journals.

The survey consisted of 27 questions covering demographics, perceptions of barriers in conservation physiology, perceptions of the success of conservation physiology and research dissemination venues and framing. The barriers we included in the survey were identified in the existing literature (Cooke and O’Connor, 2010; Caro and Sherman, 2013; Cooke *et al*., 2013; Coristine *et al*., 2014), and respondents were provided with the opportunity to add other barriers. We used a mix of Likert-style, yes/no, multiple choice and open-ended questions. The number of participants answering each question varied, and we therefore provide sample sizes for each question separately with the results. Given the breadth and number of questions we posed, we only present the data corresponding to a sub-set of the questions here. Specifically, we omitted questions asking participants about their research dissemination activities, journal choices and framing, and questions asking respondents to describe conservation physiology techniques they feel are well-validated versus those requiring more validation for application (Supplementary Information Part 2, questions 16–18, 21, 24–25), as they did not pertain to the purpose of the current manuscript (i.e. identifying the major barriers and perception of success of conservation physiology). An overview of the questions we assessed for this manuscript partitioned by topic, along with response rates, can be found in Supplementary Table 1.

Open-ended questions were manually coded thematically by the lead author to provide context to the patterns in the data. Thematic codes were determined inductively after reading all of the responses and assigned during a second reading (Thomas, 2006). We also use the open-ended question responses as a source of quotes below to better articulate some of the underlying viewpoints that the survey uncovered.

## Who is engaging in conservation physiology research?

As stated above, any individual that had experience at the intersection of physiology and conservation was permitted to complete the survey, meaning that we would obtain a range of perspectives spanning those that have only worked briefly on conservation physiology research to those whose major research focus is centred on the discipline. Our first goal was therefore to characterize the general composition of the research community contributing to the field of conservation physiology by determining where individuals work, their career stage and their taxa of study, as well as whether they consider conservation physiology to be a major disciplinary focus of theirwork.

The majority of respondents (73.6%; *n* = 345) were employed at academic institutions (university/college), 10.2% (*n* = 48) in a multi-sector capacity (mostly joint between academics and government), 9.4% (*n* = 44) at a governmental agency, with the remaining individuals (*n* = 32) employed in private sectors, research institutes or currently unemployed (Supplementary Table 2). Not surprisingly given the methodology we used to locate the participant pool, 44.3% (*n* = 207) of respondents were research faculty, with other major percentages being represented by graduate students/post-doctoral fellows (17.6%; *n* = 82), governmental scientists (13.7%; *n* = 64), educator/lecturers (9.6%; *n* = 45) and non-governmental scientists (9.0%; *n* = 42). In addition, most respondents were male (62.5%; *n* = 290). Full demographic data can be found in the Supplementary Information (Table 1). While our survey did not ask participants to provide information on the geographic regions in which they have lived, studied or worked, our sample included respondents located around the globe including North America (45%), South America (3%), Australia and New Zealand (9%), Africa (2%), eastern and western Europe (28%) and Asia (4%) (based on IP addresses, with 9% unidentified).

Respondents work on a diversity of taxa (Supplementary Figure 1) with fish (excluding elasmobranchs) comprising the research foci of 18% (*n* = 127) of participants, plants 16% (*n* = 109), mammals 15% (*n* = 106), invertebrates 14% (*n* = 98), birds 12% (*n* = 80), reptiles 9% (*n* = 64), amphibians 7% (*n* = 45) and algae 3% (*n* = 19). The remaining respondents (*n* = 47) focus on elasmobranchs (2%), bacteria (2%), fungi (1%), lichens (0.1%) or take a non-taxonomic or full ecosystem approach to their work (1%). It should be noted that some respondents work on more than one taxonomic group and percentages are calculated over total responses. We find it interesting that there was such a large constituent of respondents identifying plants and invertebrates as their taxa of focus. Generally, these taxonomic groupings tend to be under-represented in much of the conservation physiology literature (Lennox and Cooke, 2014; van Kleunen, 2014; Madliger *et al*., 2018).

Overall, one-third of the respondents (35%; *n* = 148) considered themselves to be a ‘conservation physiologist’, while the remaining two-thirds (65%; *n* = 275) did not (based on the question ‘*Do you consider yourself a Conservation Physiologist*?’). We did not see a difference in age, gender or job title/position between participants that identified as conservation physiologists versus those that did not (Supplementary Table 3), indicating that these demographic characteristics do not likely dictate entry into the field. Overall, within each taxonomic focus, <50% of researchers surveyed self-identify as conservation physiologists, with those studying fish constituting the largest absolute number of conservation physiologists. Indeed, some of the best-documented, earliest success stories in conservation physiology have been related to the management of native fishes, such as Pacific salmon (see Cooke *et al*., 2021 for a summary). Research on species of fisheries importance is often able to link more easily with conservation applications because of the long-standing policy channels accessible to fishery biologists. Both of these reasons could have led to a greater influx of individuals formulating research foci or entire research programmes on the conservation physiology of fishes.

When asked to list disciplines that their research falls under, respondents provided a diversity of fields ranging from behavioural ecology to botany to evolutionary physiology to restoration ecology (Supplementary Table 4). The field of conservation physiology being relatively new in name could mean it has lacked overall exposure. For example, one respondent who self-identified as a seagrass ecologist and ecophysiologist stated

Until being asked to participate, I was unaware that there was a field of conservation physiology. I think that my work broadly fits into this category but I had never heard it phrased in this manner. [Governmental scientist,USA]

Similarly, another participant indicated

I would be really curious to know how many of us are out there. I am the only conservation physiologist I know. [Non-governmental scientist,USA]

**Figure 1:**
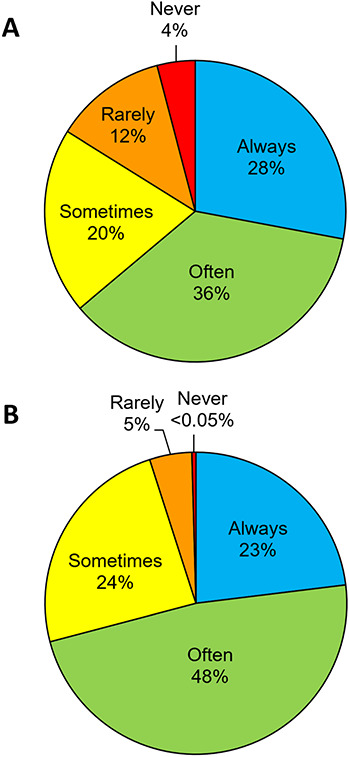
Frequency with which participants incorporate (A) physiological techniques (*n* = 465) and (B) applied conservation approaches (*n* = 464) into their current research/work.

There are also likely conservation scientists that have only briefly employed physiological approaches but have not incorporated physiology into their ongoing research programs. Indeed, 36% of respondents never (*n* = 19), rarely (*n* = 56) or only sometimes (*n* = 93) incorporate physiological techniques into their current research program (Figure 1A). Likewise, some physiologists may have collaborated on a conservation endeavour, but do not do so regularly given that 30% of respondents never (*n* = 2), rarely (*n* = 21) or only sometimes (*n* = 112) take an applied conservation approach in their work (Figure 1B). Participants who considered themselves a ‘conservation physiologist’ more often take a physiological approach to their work, and more often consider the applied implications of their work, compared to non-conservation physiologists (Supplementary Figure 2). This is not entirely surprising, as we expect conservation physiologists to be merging the two disciplines on a regular basis. While we do not believe it is necessary to self-identify as a conservation physiologist to accomplish fruitful integrations between conservation science and physiology, we believe it is useful to encourage the formation of a community of scientists that can share ideas and establish an evidence base. It is therefore possible that the field of conservation physiology is missing out on collaborations and perspectives that could encourage growth, especially if some researchers feel isolated. We provide recommendations for increasing the visibility of the discipline in our concluding section.

## How are individuals entering the field?

We aimed to determine whether educational or training experience could influence the likelihood an individual would become a conservation physiologist. We found no differences between conservation physiologists and non-conservation physiologists in regard to formal training experiences (i.e. coursework, laboratory techniques and fieldwork) in either conservation or physiology. Of respondents self-identifying as conservation physiologists, 99% (*n* = 146) had formal training in physiology and 87% (*n* = 129) had training in conservation science. With regard to non-conservation physiologists, 93% (*n* = 255) had formal training in physiology, while 82% (*n* = 226) had training in conservation science.

 We acknowledge that receiving training at the university level in a classroom setting can be very different than hands-on training. We therefore asked respondents to further indicate the type of training they received. A total of 73% (*n* = 106) of conservation physiologists identified laboratory and/or field work as part of their physiological training compared to a similar 69% (*n* = 175) of non-conservation physiologists. For conservation training, 71% (*n* = 92) of conservation physiologists indicated that they received hands-on laboratory or field training in comparison to 64% (*n* = 145) of non-conservation physiologists. Overall, these comparable proportions indicate that exposure to formal training is not likely to dictate entry into the field. However, hands-on training in conservation science may slightly increase chances of students pursuing futures in conservation physiology. It is logical that exposure to concepts in conservation physiology specifically (i.e. course sections or entire courses dedicated to conservation physiology) may influence future interest in the discipline, but this remains to be causally explored.

## Is the field of conservation physiology perceived as successful?

**Figure 2:**
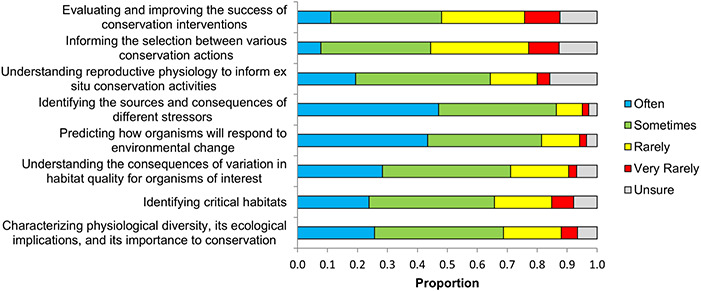
Success of conservation physiology in accomplishing its goals, as outlined in Cooke *et al*., 2013 (number of responses varied from 423 to 426).

**Figure 3:**
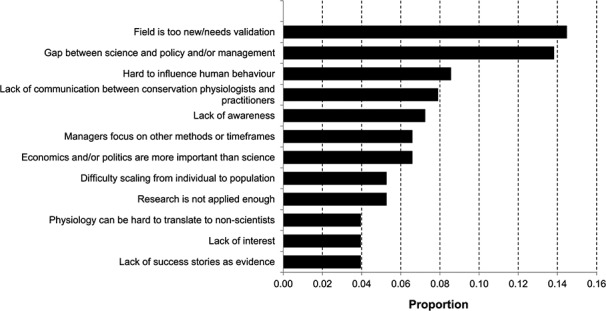
Reasons that conservation physiology may not lead to success (*n*=152), with success being defined as a change in human behaviour, management, or policy (only proportions over 0.03 are displayed).

In 2013, leaders in the field refined the definition of conservation physiology and outlined its eight primary goals (Cooke *et al*., 2013; Figure 2). The two goals that were viewed by survey respondents as most often accomplished are ‘identifying the sources and consequences of different stressors’ and ‘predicting how organisms will respond to environmental change’ (Figure 3), with 86% (*n* = 200) and 81% (*n* = 185) of respondents, respectively, indicating that the goal is accomplished ‘often’ or ‘sometimes’. In contrast, the goals that respondents felt were least often accomplished were ‘evaluating and improving the success of conservation interventions’, ‘informing the selection between various conservation actions’ and ‘understanding reproductive physiology to inform ex situ conservation activities’ (Figure 2). These three goals have the strongest ties to on-the-ground conservation efforts, indicating that respondents may view conservation physiology as needing to make more progress in achieving its ultimate goal of *solving* conservation problems (Cooke *et al*., 2013). Only a little over 1% (*n* = 6) of total respondents (*n* = 431) indicated that conservation physiology ‘very often’ leads to conservation success (defined as ‘a change in human behaviour, management or policy’). It was much more common for respondents to feel that conservation physiology sometimes (47%; *n* = 203) or rarely (43%; *n* = 186) leads to success, and a small proportion of respondents (2%, *n* = 7) felt that conservation physiology has never led to success.

Respondents who indicated that they believe conservation physiology rarely or never leads to conservation success were prompted to articulate their reasons. The responses varied, but a number of common reasons emerged (Figure 3). For example, 14% (*n* = 22) of respondents believed the field is too new or requires more validation before it can lead to measurable conservation success. One respondent stated:

I think the fields of conservation and physiology are not well integrated, e.g. we hardly ever see a physiologist at the table of our conservation workshops in which we set management priorities for the conservation of specific species. I believe many conservationists do not realize what role physiology could play in conservation. [Non-governmental scientist, The Netherlands]

A similar proportion of participants (14%; *n* = 21) believed that the gap between science and policy/management precludes findings in conservation physiology from being translated into conservation solutions. Most of these respondents indicated that the primary literature is not translated into on-the-ground action or used as a decision-making tool by agencies, or that professionals involved in environmental policy have little knowledge of physiological work or the necessary training to interpret it. Other reasons included a general difficultly in influencing human behaviour with science (9%; *n* = 13), lack of communication between conservation physiologists and practitioners (8%; *n* = 12), lack of awareness of the field of conservation physiology as a potential contributor (7%; *n* = 11) and the opinion that managers focus on other methods or timeframes apart from physiology (7%; *n* = 10) (Figure 3; Supplementary Table 5). For example, one respondent stated:

I think that readers/stakeholders still perceive physiological responses as short term, or temporary, and not really having a long term, population level effect. [Research faculty member, Canada]

Another participant articulated why managers may look towards other methodologies:

[It is] currently too easy to skip the causal mechanism (physiological response) and assume that x-environmental stressor is the [sic] responsible for observed declines in a population. [Governmental scientist, no location provided]

These responses suggest that the merits of understanding mechanism for conservation (Seebacher and Franklin, 2012), and the fact that physiological traits can be linked to the demographic processes that drive population change over longer time periods (Bergman et al. 2019), may be unclear. We believe that conservation physiologists have the willingness and power to make stronger connections and promote the value of their evidence-based science. Despite many cogent arguments available in the scientific literature on the value of using mechanistic physiological measures for determining cause–effect relationships (Carey, 2005; Pörtner and Peck, 2010; Ellis *et al*., 2011; Blaustein *et al.*, 2012; Seebacher and Franklin, 2012), we believe explicit success stories (e.g. Tracy *et al*., 2006; Cooke *et al*., 2012; Donaldson *et al*., 2013; Madliger *et al*., 2016; Madliger et al. 2021) will speak more loudly than theoretical arguments.

Interestingly, participants were comparatively more confident that their own work will result in conservation success, with 41% (*n* = 177) stating that their research is in the process of contributing to conservation success and 19% (*n* = 83) indicating that their work has already done so. It is important to note that self-reporting of successes is inherently subject to bias with potential for level of success to be inflated (van de Mortel, 2008). Of the 33% (*n* = 140) of respondents that indicated their work has not led to conservation success, the reasons varied (thematized open-ended question). The majority of respondents (40%; *n* = 56) did not provide a reason or stated that they were unsure. Approximately 17% (*n* = 24) indicated that their results did not generate actionable data for conservation science (i.e. the work was too theoretical, the research was only a small part of a larger project or the work was completed on a small scale). A similar number of respondents (15%; *n* = 21) expressed that their work was discouraged or ignored in some way by decision-makers, citing that policy is often more interested in economic interests, that policy-makers often support research that is already in line with their goals or that it would take overwhelming evidence to change existing policy. Other reasons respondents felt their work had not been translated into success included limited time (9%; *n* = 13), lack of connection to conservation practitioners (9%; *n* = 12), a feeling that physiology is not yet being accepted by conservation science (5%; *n* = 7), that they did not try (2%, *n* = 4) or that their work showed there was no conservation issue (2%, *n* = 3). Given that many participants indicated that their work is in the process of contributing to on-the-ground success, we anticipate that there could be many new opportunities to highlight the benefits of physiological approaches to conservation science in the near future. We provide further recommendations for increasing the reach and success of conservation physiology below.

## What are the challenges that prevent conservation physiology from achieving conservation success?

**Figure 4:**
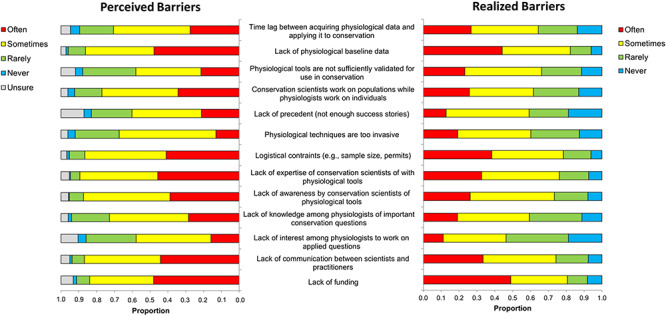
Frequency of perceived and realized barriers in conservation physiology (number of responses varied from 452 to 467).

We queried participants about the barriers they perceived to be negatively impacting the growth of the field. Nearly 50% of participants believe researchers ‘often’ face the challenges of lack of physiological baseline data (*n* = 222), lack of funding (*n* = 224), lack of expertise of conservation scientists with physiological tools (*n* = 212) and lack of communication between scientists and practitioners (*n* = 205) (Figure 4). In contrast, physiological techniques being too invasive (*n* = 61), lack of success stories (*n* = 98) and a lack of interest among physiologists to work on applied questions (*n* = 74) were perceived to be less-common problems, with fewer than 20% of participants citing them as a barrier that is ‘often’ faced (Figure 4). A key pattern, however, is that all of the barriers presented were perceived as relatively persistent, in that over half of participants indicated they were ‘often’ or ‘sometimes’ occurring (Figure 4).

**Figure 5:**
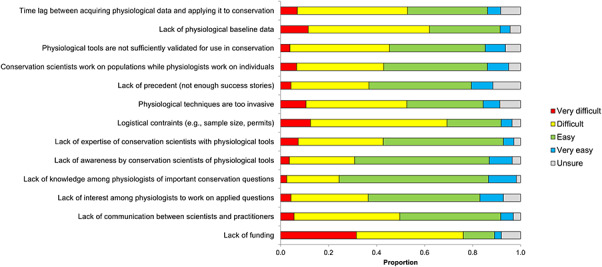
Level of difficulty associated with overcoming barriers in conservation physiology (number of responses varied from 459 to 463).

When we compare patterns in perceived barriers to realized barriers (i.e. the frequency with which participants *personally* faced the same barriers), the trends are similar (Figure 4). Again, lack of funding (*n* = 223) and lack of physiological baseline data (*n* = 202) are still experienced ‘often’ by nearly 50% of respondents. However, lack of communication among scientists and practitioners and a lack of expertise of conservation scientists with physiological tools are not faced as often as they are perceived to be. Indeed, there is a greater proportion of individuals who ‘rarely’ or ‘never’ personally face these barriers compared to a participant’s perception of what the field is generally experiencing (Figure 4). This provides some optimism, in that the actual challenges that must be overcome may be less common than imagined and we likely need greater communication even among conservation physiology researchers on where to place future effort for progressing the field.

A barrier may be frequent, but if easily overcome, it may not amount to a great impediment. As a result, we asked participants to indicate their level of difficulty in overcoming each barrier (Likert-scale) and found that, apart from lack of funding, very few respondents (<15%) viewed any barrier as ‘very difficult’ to surmount (Figure 5). However, many barriers were still considered ‘difficult’ to address, with lack of funding, logistical constraints (e.g. sample size, permits), lag time between acquiring physiological data and applying it to conservation and lack of baseline physiological data representing the most arduous barriers (Figure 5). In contrast, other limitations are viewed as more surmountable. For example, lack of awareness by conservation scientists of physiological tools, lack of knowledge among physiologists of important conservation questions and lack of interest among physiologists to work on applied questions were seen by many (>55%) as ‘easy’ or ‘very easy’ to tackle. Many researchers believed that barriers associated with knowledge or awareness can be reversed, and we advocate that this can be achieved through education or increased communication among physiologists and conservation scientists (see below).

**Figure 6:**
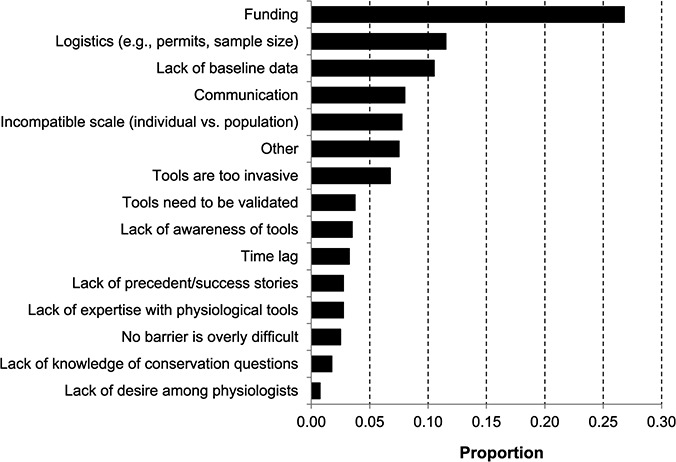
Most difficult barriers to overcome in conservation physiology (311 respondents provided 399 barriers; some participants listed two or more barriers as being equally difficult); see text for additional information on responses comprising the `other' category.

Finally, we gauged which barrier individuals have found to be the most difficult to face personally and the reason why (thematized open-ended responses). Over 26% (*n* = 107) of responses were focused on funding (Figure 6). For example, one respondent stated:

There seems to be significantly more funding for physiological research in an evolutionary context and/or research directly applicable to human health than funding available for physiology with intentional conservation applications. [Educator/lecturer, USA]

A number of respondents also indicated that they feel pressure to downplay the applied aspects of their research when applying for large grants. One participant stated:

Conservation needs to be hidden within a question that large funding bodies find more relevant. [Graduate student/post-doctoral fellow, USA]

Others mentioned that funding for conservation-focused research often necessitates working on an imperilled species, but that applying physiological tools in such species is seen as too invasive, or it is impossible to attain the necessary sample sizes for biological/statistical relevance. This speaks to the fact that some challenges are intertwined; funding (27%; *n* = 107), logistical constraints (11%; *n* = 46) and invasiveness of techniques (7%; *n* = 27) were often mentioned in combination.

Lack of physiological baseline data was identified as the most difficult barrier to overcome by 11% (*n* = 42) of participants (Figure 6). Again, there was some inter-connectedness identified between barriers as a number of participants indicated that funding to collect this type of data is hard to attain. One participant stated:

...very often in conservation the need for baseline data isn't realized until some acute problem expresses itself, at which point it is too late to collect baseline data. [Governmental scientist, USA]

And still others expressed concern that baseline data often must come from proxies. For example, one respondent indicated:

[Physiological baseline data] comes predominantly from more lab-friendly model species, which tend not to be those of conservation interest, and are often so taxonomically different that extrapolation of the baseline data is speculative... This makes it very difficult to interpret the physiological data from the species of interest sufficiently robustly that it can be confidently applied to conservation actions. [Graduate student/post-doctoral fellow, Finland]

Approximately 8% (*n* = 30) of respondents identified a barrier that was not provided in the survey (‘Other’ in Figure 6). Half of these pointed to lack of interest in conservation physiology among conservation scientists as the most difficult barrier they have faced. Participants cited a number of reasons that appear to account for the lack of interest, including that conservation scientists do not believe physiological tools are useful, have unreasonable expectations of sample sizes or that the tools appear confusing or expensive (in some cases because physiologists have trouble showing or explaining how their tools can be useful). Related to this, another barrier that came up repeatedly (*n* = 7) was that conservation scientists and physiologists lack an understanding of one another’s disciplines, having different underlying priorities, concerns, histories and viewpoints and, occasionally, a lack of respect for one another’s disciplines. In many ways, this is symptomatic of working in interdisciplinary fields. Interdisciplinarity, especially in the conservation sciences, is absolutely essential (Dick *et al*., 2016), yet there are many challenges to doing so (Rhoten and Parker, 2004). However, there are a number of proactive strategies for overcoming the barriers that were reinforced by some of the survey respondents and also previously discussed in a reflective article on conservation physiology in practice as it relates to Pacific salmon (Cooke *et al*., 2012).

## Twelve suggested actions for overcoming barriers in conservation physiology

**Figure 7:**
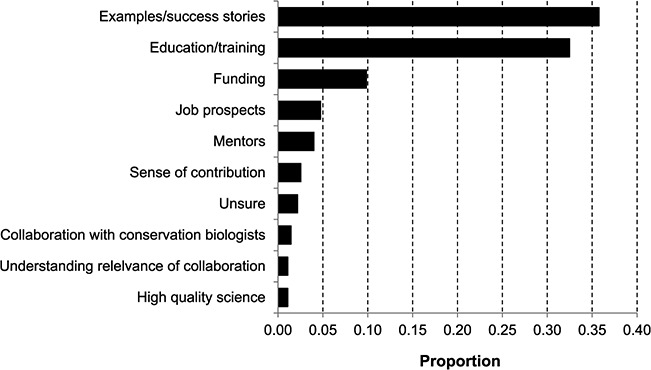
Suggestions for encouraging a new generation of students to become interested in conservation physiology (*n* = 274). Only responses that were provided by more than one individual are displayed; remaining responses accounted for 5% of the total.

**Figure 8:**
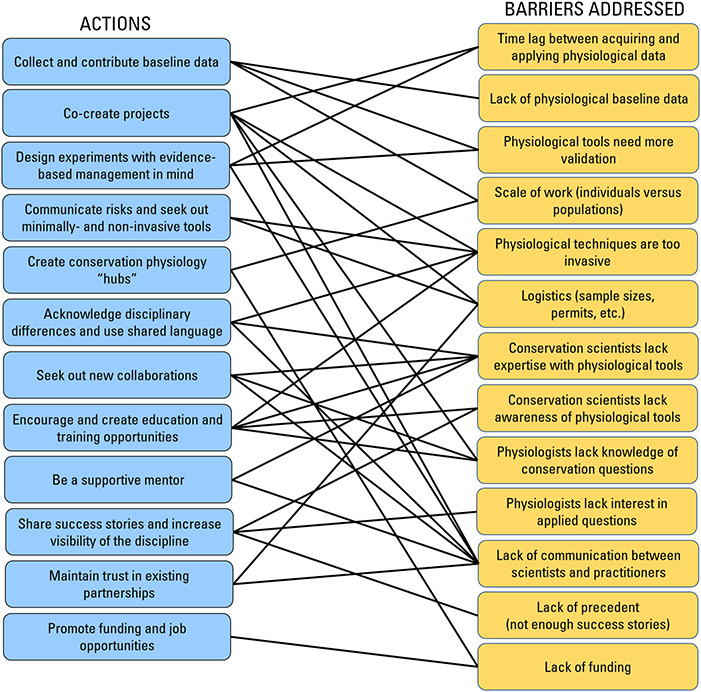
Diagram illustrating which barriers in conservation physiology can be addressed by each of the 12 proposed actions.

We conceptualized the following 12 actions based on our own experiences, by considering the barriers identified above and through feedback we received from survey respondents regarding what they feel is needed to inspire an up-coming generation of biologists to consider becoming conservation physiologists (Figure 7, thematized open-ended question; Figure 8). In particular, respondents indicated that success stories, education/training, funding, job prospects and inspiring mentors are most needed to raise the profile of conservation physiology in the minds of students and young professionals (Figure 7). Together, these action items also attempt to address the challenges related to funding acquisition, logistics, communication, lack of knowledge/awareness, baseline data and time lags that many respondents cited as the most difficult barrier they have attempted to overcome (Figure 8).

*(1) Share success stories and increase the visibility of the discipline*: Be vocal across news media, social media, personal websites and blogs, conferences, invited lectures at institutions and government facilities, public outreach events and traditional publications with success stories in conservation physiology. Propose symposia and workshops at both physiological/integrative biology and conservation conferences that highlight how physiological approaches have helped to address conservation challenges (Madliger *et al*., 2017). Conservation physiology special issues can also be proposed to journals with a readership interested in integrative techniques for conservation science. By focusing on ‘bright spots’ where policy/practice has been successfully influenced by conservation physiology approaches, we can promote an optimistic outlook that can inspire action, promote team collaboration and coordination and support creativity in addressing challenges (Cvitanovic and Hobday, 2018).

*(2) Create conservation physiology ‘hubs’:* Following from a need to increase the visibility of the discipline, we suggest that researchers begin amalgamating their conservation physiology networks into ‘hubs’ (*sensu*  Taylor *et al*., 2017) with shared properties (e.g. taxonomic focus, sub-discipline of physiology, conservation challenge of interest). Having core groups of experts on given topics could increase the accessibility of conservation physiology techniques for managers and practitioners who are keen to begin collaborating. In addition, this type of action could provide opportunities for collaborative grants that span geography, ecosystem type and taxonomy, potentially attracting large-scale funding that could not be obtained by projects or laboratories in isolation. We also see the possibility of such collaborative groups being active on social media to share their conservation physiology work with a broader public.

The process of creating of such hubs could be well-suited to some granting programs, such as the National Science Foundation’s Research Coordination Networks funding, which supports projects that ‘advance a field or create new directions in research or education by supporting groups of investigators to communicate and coordinate their research, training and educational activities across disciplinary, organizational, geographic and international boundaries’ (National Science Foundation, 2021). As the discipline grows, there may also be the possibility to create a universal ‘hub’ in the form of an online repository that draws on knowledge and experience across the entirety of the field. Within such a repository, both academic and non-academic users could access a full index of conservation physiology literature, search for research based on a topic, generate and contribute to shared ideas, engage in discussion or pose questions to the community, identify research gaps, locate other researchers with similar interests and find new collaborations with other researchers or practitioners (e.g. Veterans Research Hub: Cooper, 2016).

*(3) Encourage education and training opportunities:* Exposure to the diversity of unanswered questions in conservation physiology can inspire curiosity and passion. Those teaching courses in conservation science and wildlife management have the ability to expose students to physiological approaches, just as those teaching animal physiology classes have the capacity to expose students to the connections that physiology can make to conservation science. In particular, exposing students to conservation physiology early in their undergraduate studies could stimulate more students to choose courses in both topics moving forward, gaining expertise that will allow them to combine tools and theory more effectively in professional settings. As the field continues to grow, we see the opportunity for upper-year undergraduate or graduate courses dedicated entirely to the topic of conservation physiology (Madliger *et al*., 2021).

*(4) Seek out new collaborations:* Physiologists seeking to apply their tools more directly to conservation initiatives could begin by looking in their own backyard, contacting (i) fellow faculty members working in conservation science to brainstorm collaborative opportunities; (ii) local conservation authorities (near their institution or field sites) to understand their mandate, as well as the specific challenges they are working on addressing; and (iii) local government agencies that focus on wildlife management to inquire about the opportunity to present their work and the value of their tools.

*(5) Maintain existing partnerships:* While the process of building and maintaining trusting relationships may require a non-trivial time investment, it is well-established that it is a critical component for meaningful information and knowledge exchange (Jacobs *et al*., 2005; Gibbons *et al.*, 2008; Roux *et al*., 2006; Young *et al.*, 2014). For example, workshops, field trips, secondments, fellowships and sabbaticals can represent opportunities to focus on bridging the gap between physiology and conservation science and maintaining connections (Gibbons *et al*., 2008). This continued contact will act to build trust and respect between all parties, which is considered integral to interdisciplinary success (Daily and Ehrlich, 1999; Chapman *et al*., 2015). If honest, transparent partnerships are maintained, even outside of the timeframe of targeted projects, it is likely that additional opportunities for informing management will develop (Brooks *et al*., 2018).

*(6) Acknowledge disciplinary differences and develop a shared language:* Disciplinary differences between physiologists and conservation scientists can undoubtedly create barriers in communication. However, it is this diversity of opinion, techniques and history that can spark innovative approaches to addressing conservation challenges. Be open and honest about lack of expertise and use collaborations as an opportunity to grow and find a shared language (Bracken and Oughton, 2006). The act of establishing a shared goal can be a reasonable first step in streamlining communication, and we suggest openly sharing any reservations, such as the level of invasiveness of techniques, timeframes, costs and sample sizes. This type of dialogue is essential to dispelling misconceptions and gaining the trust necessary for long-term interdisciplinary partnerships (Pooley *et al*., 2014; Dick *et al*., 2016).

*(7) Co-create projects:* Conservation physiologists need to become involved early-on in targeted projects where their physiological tools can have relevance (i.e. co-create success stories with practitioners and managers) (Brooks *et al*., 2018; Laubenstein and Rummer, 2021). Indeed, the idea of co-creation of the research agenda and co-production of knowledge are regarded as fundamental to achieving success in applied realms such as conservation science (Colloff *et al*., 2017). The conservation toolbox is quite vast (Madliger *et al*., 2018), and it will be through careful planning at the onset of projects that data will become useful for management purposes (Fazey *et al*., 2013; Clark *et al*., 2016).

*(8) Design experiments with evidence-based management in mind:* The systematic review of accumulated evidence is becoming a growing part of environmental management decisions (Sutherland *et al*., 2004; Pullin and Knight, 2009; Cooke *et al*., 2017b). Conservation physiologists can contribute to available evidence bases by ensuring their empirical work is included in systematic reviews (Cooke *et al*., 2017a). Carefully planning sample sizes, using replicates, acknowledging bias, using controls and reporting descriptive data are all essential to formulating a study that can contribute to evidence synthesis (Cooke *et al*., 2017a).

*(9) Be a supportive mentor:* Provide mentees with connections to other students and professionals working in the field, opportunities to connect with on-the-ground projects and skill-building workshops. Connect students to your own current and past mentors and other pioneers in the field. Overall, promote diversity and an environment where students of any identity or background feel supported and included (Gould *et al*., 2018).

*(10) Promote funding and job opportunities:* As a scientific community, we have never been more connected, in large part due to social media. With this comes the opportunity to share successes and challenges that can assist the conservation physiology community in identifying where and how to apply for funding, as well as job postings that are relevant to conservation physiologists. When proposing large, collaborative grants, consider how physiological investigations could simultaneously be valuable in both pure and applied contexts.

*(11) Communicate risks and seek out minimally invasive and non-invasive alternatives where appropriate:* It is possible that many physiological techniques are viewed as more invasive than they truly are in practice, and it will therefore be important for physiologists to take time to communicate the details of methodologies when approaching new collaborations. Just as important, conservation scientists beginning to work with physiologists should communicate the acceptable degree of animal handling and anticipated sample sizes. In many cases, there may be minimally invasive options or physiological measurements that can be taken in conjunction with other data already requiring animals to be handled.

*(12) Contribute baseline data:* Since conservation physiology was formally conceptualized, lack of baseline data has been outlined as an impediment to employing physiology as a conservation monitoring tool (Wikelski and Cooke, 2006). However, physiological data have been accumulating for many species, including information on both inter- and intra-individual variation (e.g. how physiology changes with development and age, reproductive states, seasons), which is essential for accurately interpreting results and providing recommendations based on physiological changes over time or between populations. For example, HormoneBase is a recently launched, freely accessible online database of over 6500 glucocorticoid and androgen measurements taken in adult vertebrates (Vitousek *et al*., 2018) that continues to grow. We anticipate that the expansion of existing databases or creation of new databases for other physiological traits could become similarly well-populated, and we urge researchers, managers, and wildlife veterinarians to commit their data to these pursuits.

## Conclusion

Identifying the challenges faced by researchers integrating conservation science and physiology represents the first step in working collaboratively to find solutions. While some barriers will likely prove more difficult to solve in the short-term (e.g. funding), we believe the growth in interdisciplinarity across all facets of conservation science (see Dick *et al*., 2016) will open more doors for conservation physiology. In particular, the current cohort of graduate students and early career researchers will have much to be optimistic about as the list of success stories and dedicated mentors and educators in conservation physiology continues to grow. The 12 actions for overcoming conservation physiology challenges identified here (Figure 8) are exclusively from the perspective of the ‘researcher’. There is also much that could be learned from conducting a survey of practitioners to better understand their perspectives on conservation physiology—not unlike a recent study conducted on conservation genomics (see Kadykalo *et al*., 2019). Moreover, there would be great value in investigating how geographic location (e.g. where individuals have studied, been employed and/or completed field or laboratory studies) or their level of local or international collaboration has impacted their experience in the field. Since our study at the outset was designed to be exploratory, we have been unable here to test explicit hypotheses. However, this work provides an important foundation for future hypothesis-driven quantitative studies—something we strongly encourage as the next productive steps to move this field forward.

## Supplementary Material

Supplementary_Material_Madliger_et_al_CP_Survey_coab030
