# Supplementary material for: Researcher perspectives on challenges and opportunities in conservation physiology revealed from an online survey
Source: Conserv Physiol. 2021 Apr 28;9(1):coab030. doi: 10.1093/conphys/coab030 (PMC8084030; doi:10.1093/conphys/coab030)
Supplement: Supplementary_Material_Madliger_et_al_CP_Survey_coab030 [file supplementary_material_madliger_et_al_cp_survey_coab030.docx]

**Supplementary Information**

**Part 1:** Search strings used to identify papers authored by researchers that combine physiology and conservation science (search completed on December 1, 2016).

A) To identify publications covering physiological topics in conservation journals:

Topic: (physiol* OR ecophysiol* OR hormon* OR immun* OR epidem* OR stress* OR oxidative OR endocrin* OR energetic OR neurophys* OR cort* OR gluco* OR nutrit* OR metab* OR respir* OR sensory OR photosynthe* OR "gas exchange") AND (Publication name: AMBIO OR “ANIMAL CONSERVATION” OR “APPLIED VEGETATION SCIENCE” OR “AQUATIC CONSERVATION-MARINE AND FRESHWATER ECOSYSTEMS” OR “AVIAN CONSERVATION AND ECOLOGY” OR “BIODIVERSITY AND CONSERVATION” OR “BIOLOGICAL CONSERVATION” OR “BIOLOGICAL INVASIONS” OR “CONSERVATION BIOLOGY” OR “CONSERVATION EVIDENCE” OR “CONSERVATION LETTERS” OR “CONSERVATION PHYSIOLOGY” OR “DIVERSITY AND DISTRIBUTIONS” OR “ENDANGERED SPECIES RESEARCH” OR “ENVIRONMENTAL CONSERVATION” OR “ENVIRONMENTAL MANAGEMENT” OR “FISHERIES RESEARCH” OR “FOREST ECOLOGY AND MANAGEMENT” OR “FRONTIERS IN ECOLOGY AND THE ENVIRONMENT” OR “GLOBAL CHANGE BIOLOGY” OR “JOURNAL OF APPLIED ECOLOGY” OR “JOURNAL OF WILDLIFE MANAGEMENT” OR ORYX OR “RESTORATION ECOLOGY” OR “TROPICAL CONSERVATION SCIENCE”)

Retained full 3,287 results

B) To identify publications covering conservation topics in physiological journals:

Topic: (conservation OR management OR endangered OR threat* OR invasive OR translocat* OR "captive breeding" OR biodivers* OR restoration OR "biological diversity" OR extinct* OR recovery OR policy OR "climate change") AND Publication name: (“COMPARATIVE BIOCHEMISTRY AND PHYSIOLOGY A-MOLECULAR & INTEGRATIVE PHYSIOLOGY” OR “COMPARATIVE BIOCHEMISTRY AND PHYSIOLOGY B-BIOCHEMISTRY & MOLECULAR BIOLOGY” OR “GENERAL AND COMPARATIVE ENDOCRINOLOGY” OR “HORMONES AND BEHAVIOR” OR “INTEGRATIVE AND COMPARATIVE BIOLOGY” OR “JOURNAL OF APPLIED PHYSIOLOGY” OR “JOURNAL OF COMPARATIVE PHYSIOLOGY B-BIOCHEMICAL SYSTEMIC AND ENVIRONMENTAL PHYSIOLOGY” OR “JOURNAL OF EXPERIMENTAL BIOLOGY” OR “JOURNAL OF EXPERIMENTAL ZOOLOGY PART A-ECOLOGICAL GENETICS AND PHYSIOLOGY” OR “JOURNAL OF PLANT PHYSIOLOGY” OR “PHYSIOLOGICAL AND BIOCHEMICAL ZOOLOGY” OR “PHYSIOLOGY & BEHAVIOR” OR “PLANT PHYSIOLOGY” OR “TREE PHYSIOLOGY”)

The following non-wildlife categories were excluded : SPORTS SCIENCES and PSYCHOLOGY BIOLOGICAL

Retained top 3,000 results sorted by relevance

C) To identify publications covering physiology and conservation topics simultaneously in general ecology journals:

Topic: (conservation OR management OR endangered OR threat* OR invasive OR translocat* OR "captive breeding" OR biodivers* OR restoration OR "biological diversity" OR extinct* OR recovery OR policy OR "climate change") AND (physiol* OR ecophysiol* OR hormon* OR immun* OR epidem* OR stress* OR oxidative OR endocrin* OR energetic OR neurophys* OR cort* OR gluco* OR nutrit* OR metab* OR respir* OR sensory OR photosynthe* OR "gas exchange") AND Publication name: (“ACTA OECOLOGICA-INTERNATIONAL JOURNAL OF ECOLOGY” OR “ADVANCES IN ECOLOGICAL RESEARCH” OR “ANIMAL BEHAVIOUR” OR “BASIC AND APPLIED ECOLOGY” OR “BEHAVIORAL ECOLOGY” OR “BEHAVIORAL ECOLOGY AND SOCIOBIOLOGY” OR “BIOLOGY LETTERS” OR BIOSCIENCE OR BIOTROPICA OR BOTANY OR “COMMUNITY ECOLOGY” OR ECOGRAPHY OR “ECOLOGICAL APPLICATIONS” OR “ECOLOGICAL ENTOMOLOGY” OR “ECOLOGICAL INDICATORS” OR ECOLOGY OR “ECOLOGY AND EVOLUTION” OR “ECOLOGY LETTERS” OR “ECOLOGY OF FRESHWATER FISH” OR FLORA OR “FOREST SCIENCE” OR “FRESHWATER BIOLOGY” OR “FRESHWATER SCIENCE” OR “FRONTIERS IN PLANT SCIENCE” OR “FUNCTIONAL ECOLOGY” OR “FUNGAL ECOLOGY” OR “GLOBAL ECOLOGY AND BIOGEOGRAPHY” OR “JOURNAL OF ANIMAL ECOLOGY” OR “JOURNAL OF AVIAN BIOLOGY” OR “JOURNAL OF BIOGEOGRAPHY” OR “JOURNAL OF ECOLOGY” OR “JOURNAL OF EXPERIMENTAL MARINE BIOLOGY AND ECOLOGY” OR “JOURNAL OF MAMMALOGY” OR “JOURNAL OF ORNITHOLOGY” OR “JOURNAL OF PLANKTON RESEARCH” OR “JOURNAL OF PLANT ECOLOGY” OR “JOURNAL OF VEGETATION SCIENCE” OR “JOURNAL OF WILDLIFE DISEASES” OR “LANDSCAPE ECOLOGY” OR “MARINE BIOLOGY” OR “MARINE ECOLOGY PROGRESS SERIES” OR “MICROBIAL ECOLOGY” OR NATURE OR “NEW PHYTOLOGIST” OR “OECOLOGIA” OR OIKOS OR “PLANT ECOLOGY” OR “PLANT ECOLOGY & DIVERSITY” OR “PLOS ONE” OR “PROCEEDINGS OF THE ROYAL SOCIETY B-BIOLOGICAL SCIENCES” OR SCIENCE OR “AMERICAN NATURALIST” OR “TRENDS IN ECOLOGY & EVOLUTION” OR “TROPICAL ECOLOGY” OR “WILDLIFE RESEARCH”)

Retained top 3,000 results sorted by relevance

D) To identify explicit conservation physiology publications:

Topic: "conservation physiology"

Retained all 134 results

**Part 2:** Survey instrument

**
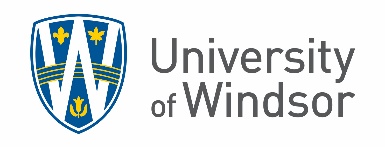

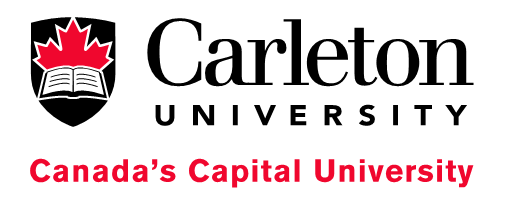
**

**Title of Study**: Scientific perspectives on realized and perceived challenges in Conservation Physiology

Thank you for your interest in participating in our research investigating the current state and future of the discipline of Conservation Physiology. Our study focuses on barriers to the integration of conservation and physiology, experiences disseminating research, and whether the field of Conservation Physiology is currently accomplishing its primary goals. Your responses will help us to better understand how the discipline can best contribute to on-the-ground conservation success.

This online survey should take 20 minutes to complete. It contains two sections, one dealing with demographics and work experience, and the other focused on the barriers, status, and future of Conservation Physiology.

The lead researcher for this study is Dr. Christine Madliger, a post-doctoral researcher in the Department of Biological Sciences at the University of Windsor. She is co-supervised by Dr. Steven Cooke from the Fish Ecology and Conservation Physiology Laboratory at Carleton University and Dr. Oliver Love in the Department of Biological Sciences at the University of Windsor.

Participating in this research is voluntary. You may withdraw from the study at any time prior to hitting the "submit" button by simply exiting the survey. While complete responses are the most useful, you have the right to skip any question(s). Your responses are anonymous and will not be linked in any way to your e-mail address or any other component of your identity. We also ask that you avoid providing any identifying information in the survey to maintain this anonymity. Overall, we do not anticipate that participating in this survey poses any psychological or physical risks. In addition, no monetary compensation will be provided in exchange for your participation.

All data generated will be stored in a password-protected computer. The company hosting the survey, Fluid Surveys, is based in Canada and responses will be stored on the company's Canadian servers. Research data will be accessible by the researcher, the research supervisors, and the survey company. Information from the survey will be reported in aggregate. The data generated from the survey will be retained by the lead researcher to potentially be used for other research projects on similar topics, in publications, and in presentations.

You have the right to request a copy of the finished research project. To do so, please contact the lead researcher.

This project was reviewed and cleared by the University of Windsor Research Ethics Board (REB). If you have any questions about the research or the associated procedures, please contact the lead researcher (contact information below). Questions regarding your rights as a participant can be directed to the REB.

**Research Ethics Board (REB) contact information:**

Research Ethics Coordinator

University of Windsor

401 Sunset Avenue

Windsor, Ontario, Canada, N9B 3P4

Tel: 519-253-3000 x3948

ethics@uwindsor.ca

**Lead researcher contact information:**

Dr. Christine Madliger

Department of Biological Sciences

University of Windsor

Tel: 519-253-3000 x2701

Email: madlige@uwindsor.ca

**Supervisor contact information:**

Dr. Steven Cooke

Department of Biology

Carleton University

Tel: 613-520-2600 x2143

Email: steven.cooke@carleton.ca

**By proceeding to the survey you acknowledge that you are 18 years or older, have read the statements above, and consent to participate in the research as described. You may print this page for your records.**

Have you ever participated in research or other work that combines physiology and conservation?

- No
- Yes

*If respondents choose yes, they will continue on to the survey. If respondents choose 'No', they will receive the following message:

Thank you very much for your interest in our survey. However, the target population consists of researchers and/or practitioners that have some experience combining physiological techniques and conservation. We have therefore ended the session based on your previous response. Thank you for your time and we apologize for any inconvenience.

**For the purpose of this survey, we use the following definition of Conservation Physiology, presented in Cooke et al. (2013) *Cons Phys* 1 doi: 10.1093/conphys/cot001:** An integrative scientific discipline applying physiological concepts, tools, and knowledge to characterizing biological diversity and its ecological implications; understanding and predicting how organisms, populations, and ecosystems respond to environmental change and stressors; and solving conservation problems across the broad range of taxa (i.e., including microbes, plants, and animals).

PART 1 - Demographics and Work Experience

1. Which of the following describes the sector in which you are employed or associated with (please check all that apply):

- Governmental agency
- University/college
- Not-for-profit/non-governmental
- Private sector
- Other - please specify

2. I can best be described as (please choose one):

- Governmental scientist
- Non-governmental scientist
- Private sector scientist
- Research faculty
- Educator/lecturer
- Consultant
- Manager/administrator
- Graduate student or post-doctoral fellow
- Technician/research assistant
- Other - please specify

3. My work primarily focuses on (choose all that apply):

- Algae
- Amphibians
- Bacteria
- Birds
- Elasmobranchs
- Fish
- Fungi
- Invertebrates
- Mammals
- Plants
- Reptiles
- Other - please specify

4. Do you have any formal (i.e., directed or hands-on) training in physiology? Please check all that apply.

- Yes - university coursework
- Yes - field techniques
- Yes - lab or veterinary techniques
- Yes - graduate thesis (completed or in progress)
- None

5. Do you have any formal (i.e., directed or hands-on) training in conservation biology? Please check all that apply.

- Yes - university coursework
- Yes - field techniques
- Yes - lab or veterinary techniques
- Yes - graduate thesis (completed or in progress)
- None

6. In your current position, how often do you employ physiological techniques?

- Always
- Often
- Sometimes
- Rarely
- Never

7. In your current research or work, how often do you take an applied approach (i.e., how often do you present your work as having relevance to conservation)?

- Always
- Often
- Sometimes
- Rarely
- Never

8. What is the highest degree you have earned?

- Highschool
- College or trades certificate
- Undergraduate degree
- Masters
- PhD

9. How long have you been working professionally in the science sector?

- 0 years - I am an undergraduate student
- 0 years - I am a graduate student
- Less than 5 years
- 5-10 years
- 10-20 years
- More than 20 years

10. What is your gender?

- Female
- Male
- Non-binary
- Prefer not to say

11. What is your age?

- <20
- 21-30
- 31-40
- 41-50
- 51-60
- 60+
- Prefer not to say

PART 2 - Barriers, Status, and Future of Conservation Physiology

12. The following list represents potential barriers to the integration of conservation and physiology. Please indicate how often you believe researchers/practitioners face each barrier while attempting to accomplish the goals of Conservation Physiology.

|  | Often | Sometimes | Rarely | Never | Unsure |
| --- | --- | --- | --- | --- | --- |
| Lack of funding for conservation physiology research |  |  |  |  |  |
| Lack of communication between scientists and practitioners |  |  |  |  |  |
| Lack of desire/interest among physiologists to work on applied questions |  |  |  |  |  |
| Lack of knowledge among physiologists of important questions in conservation |  |  |  |  |  |
| Lack of awareness by conservation biologists of physiological tools |  |  |  |  |  |
| Lack of expertise of conservation biologists with physiological tools |  |  |  |  |  |
| Logistical constraints (e.g., sample size, permits) |  |  |  |  |  |
| Physiological techniques are too invasive for use in conservation |  |  |  |  |  |
| Lack of precedent (not enough success stories) |  |  |  |  |  |
| Conservation biologists work on populations while physiologists work on individual (or lower) scales (top-down versus bottom-up mismatch) |  |  |  |  |  |
| Physiological tools are not sufficiently validated for use in conservation settings |  |  |  |  |  |
| Lack of physiological baseline data |  |  |  |  |  |
| Time lag between acquiring physiological data and applying it to conservation |  |  |  |  |  |
| Other - please specify |  |  |  |  |  |

13. Please indicate how easy you believe each of the following barriers to the integration of conservation and physiology are to overcome.

|  | Very difficult | Difficult | Easy | Very easy | Unsure |
| --- | --- | --- | --- | --- | --- |
| Lack of funding for conservation physiology research |  |  |  |  |  |
| Lack of communication between scientists and practitioners |  |  |  |  |  |
| Lack of desire/interest among physiologists to work on applied questions |  |  |  |  |  |
| Lack of knowledge among physiologists of important questions in conservation |  |  |  |  |  |
| Lack of awareness by conservation biologists of physiological tools |  |  |  |  |  |
| Lack of expertise of conservation biologists with physiological tools |  |  |  |  |  |
| Logistical constraints (e.g., sample size, permits) |  |  |  |  |  |
| Physiological techniques are too invasive for use in conservation |  |  |  |  |  |
| Lack of precedent (not enough success stories) |  |  |  |  |  |
| Conservation biologists work on populations while physiologists work on individual (or lower) scales (top-down versus bottom-up mismatch) |  |  |  |  |  |
| Physiological tools are not sufficiently validated for use in conservation settings |  |  |  |  |  |
| Lack of physiological baseline data |  |  |  |  |  |
| Time lag between acquiring physiological data and applying it to conservation |  |  |  |  |  |
| Other - please specify |  |  |  |  |  |

14. Please indicate how often you have **personally** faced each of the following barriers when attempting to integrate physiology and conservation.

|  | Often | Sometimes | Rarely | Never |
| --- | --- | --- | --- | --- |
| Lack of funding for conservation physiology research |  |  |  |  |
| Lack of communication between scientists and practitioners |  |  |  |  |
| Lack of desire/interest among physiologists to work on applied questions |  |  |  |  |
| Lack of knowledge among physiologists of important questions in conservation |  |  |  |  |
| Lack of awareness by conservation biologists of physiological tools |  |  |  |  |
| Lack of expertise of conservation biologists with physiological tools |  |  |  |  |
| Logistical constraints (e.g., sample size, permits) |  |  |  |  |
| Physiological techniques are too invasive for use in conservation |  |  |  |  |
| Lack of precedent (not enough success stories) |  |  |  |  |
| Conservation biologists work on populations while physiologists work on individual (or lower) scales (top-down versus bottom-up mismatch) |  |  |  |  |
| Physiological tools are not sufficiently validated for use in conservation settings |  |  |  |  |
| Lack of physiological baseline data |  |  |  |  |
| Time lag between acquiring physiological data and applying it to conservation |  |  |  |  |
| Other - please specify |  |  |  |  |

15. Which of the barriers from the previous question has been the most difficult for you to overcome and why do you believe it has been so difficult?

______________________________________________________________________________

16. How often do you disseminate your Conservation Physiology research through the following sources?

|  | Often | Sometimes | Rarely | Never |
| --- | --- | --- | --- | --- |
| News/media reports (print, television, radio, press releases, etc.) |  |  |  |  |
| Peer-reviewed publications |  |  |  |  |
| Publications or events for industry, NGOs, or other public interest groups |  |  |  |  |
| Personal contacts |  |  |  |  |
| Government websites |  |  |  |  |
| Meetings with government/stakeholder groups |  |  |  |  |
| Professional conferences |  |  |  |  |
| Personal website |  |  |  |  |
| Social media (Facebook, Twitter, LinkedIn, blogs, etc.) |  |  |  |  |
| Other - please specify |  |  |  |  |

17. If you disseminate your conservation physiology work in peer-reviewed journals, what subject matter (scope) of journal do you **primarily** choose:

- Conservation biology
- Conservation physiology
- Ecology
- Evolutionary biology
- Genetics
- Methods and techniques
- Physiology
- Taxa-specific (e.g., Journal of Avian Biology, Journal of Fish Biology)
- Broad subject matter (e.g., Proceedings of the National Academy of Sciences, Plos One)
- Other - please specify
- Not applicable

18. Do you ever feel as though you need to alter the message or framing of your Conservation Physiology research to allow it to be published in peer-reviewed journals?

- Yes, and more often compared to other research I conduct
- Yes, equivalently compared to other research I conduct
- Yes, and less often compared to other research I conduct
- No
- Not applicable

a. If you answered yes, why do you believe this is the case?

­­­­­­­­­­­­­­­­­­­­________________________________________________________________________

19. The following are the goals that Conservation Physiology aspires to accomplish (as outlined in Cooke et al. 2013 - doi: 10.1093/conphys/cot001). Please indicate how often you believe the field currently accomplishes each goal.

|  | Often | Sometimes | Rarely | Very rarely | Unsure |
| --- | --- | --- | --- | --- | --- |
| Characterizing physiological diversity, its ecological implications, and its importance for conservation |  |  |  |  |  |
| Identifying critical habitats |  |  |  |  |  |
| Understanding the consequences of variation in habitat quality for organisms of interest |  |  |  |  |  |
| Predicting how organisms will respond to environmental change |  |  |  |  |  |
| Identifying the sources and consequences of different stressors |  |  |  |  |  |
| Understanding reproductive physiology to inform ex situ conservation activities |  |  |  |  |  |
| Informing the selection between various conservation actions (i.e., choosing the conservation action that has the greatest chance of success) |  |  |  |  |  |
| Evaluating and improving the success of conservation interventions (i.e., monitoring and managing the success of conservation actions post-implementation) |  |  |  |  |  |

20. Has your work combining physiology and conservation been translated into conservation success (i.e., a measurable change in human behaviour, management, or policy)?

- Yes
- In progress
- No
- Not applicable

a. If you answered no, please briefly describe why you believe this is the case.

­­­­­­­­­­­­­­­­­­_______________________________________________________________________

21. Is there an example of work (your own or otherwise) that you believe has been particularly successful in using physiology to foster conservation success? If so, please briefly describe the work here and why you believe it has been successful.

____________________________________________________________________________

22. Overall, how often do you believe the field of Conservation Physiology influences human behaviour, management, and/or policy?

- - Very often
  - Often
  - Sometimes
  - Rarely
  - Never

a. If you believe that Conservation Physiology influences human behaviour, management, and/or policy **very often, often, or sometimes,** please indicate the primary way(s) you believe it does so.

_______________________________________________________________________

b. If you believe that Conservation Physiology **rarely or never** influences human behaviour, management, and/or policy, please indicate the primary reason(s) why you believe this is the case.

________________________________________________________________________

23. Which of the following do you think is most important for a researcher to be successful in Conservation Physiology (i.e., to allow physiological techniques to solve conservation problems)? Please choose one option.

- - technical skills in physiology
  - connection to physiology professionals
  - expertise in a specific ecosystem or species
  - connections with conservation professionals
  - strong publication record
  - access to funding
  - a personal dedication to contribute to conservation goals
  - freedom in designing a research program
  - other - please specify

a. Please describe why you believe this characteristic is the most important.

__________________________________________________________________________

24. Please provide at least one method in Conservation Physiology that you believe has the greatest capacity to solve conservation problems and why/how so. It can be a technique (e.g., telemetry) or a physiological variable or endpoint (e.g., oxidative stress).

__________________________________________________________________________

25. Please provide at least one method in Conservation Physiology that you believe needs further validation prior to use in solving conservation problems and why/in what way. It can be a technique (e.g., telemetry) or a physiological variable or endpoint (e.g., oxidative stress).

__________________________________________________________________________

26. What is most needed to inspire or allow the next generation of biologists (i.e., students currently being trained in biology) to subscribe to Conservation Physiology?

____________________________________________________________________________

27. Do you consider yourself a "Conservation Physiologist"?

- No
- Yes

a. If you answered 'no', please indicate the discipline that your work best falls under.

__________________________

b. If you answered 'yes', please indicate any other disciplines with which you also self- identify.

__________________________

If you have any additional comments or concerns regarding this survey, please provide them here.

____________________________________________________________________________

**Supplementary Table 1:** Summary of purpose/topic theme of survey questions and associated number of responses. Please see survey instrument above for full question. Questions with ranges of numbers of responses were multi-part. Full participant pool was 468 individuals.

| **Topic of interest** | **Question** | **Type of question** | **Number of responses** |
| --- | --- | --- | --- |
| Composition of conservation physiology community | Which of the following describes the sector in which you are employed or associated with (please check all that apply) | Multiple choice | 468 |
|  | I can best be described as (please choose one) | Multiple choice | 468 |
|  | What is the highest degree you have earned? | Multiple choice | 468 |
|  | How long have you been working professionally in the science sector? | Multiple choice | 468 |
|  | What is your gender? | Multiple choice | 468 |
|  | What is your age? | Multiple choice | 468 |
|  | My work primarily focuses on (choose all that apply) | Multiple choice | 468 |
|  | In your current position, how often do you employ physiological techniques? | Likert scale | 468 |
|  | In your current research or work, how often do you take an applied approach (i.e., how often do you present your work as having relevance to conservation)? | Likert scale | 468 |
|  | Do you consider yourself a "Conservation Physiologist"? | Binary choice | 423 |
|  | If you answered 'no', please indicate the discipline that your work best falls under. | Open-ended | 252 |
|  | If you answered 'yes', please indicate any other disciplines with which you also self-identify. | Open-ended | 127 |
| Training background | Do you have any formal (i.e., directed or hands-on) training in physiology? Please check all that apply. | Multiple choice | 468 |
|  | Do you have any formal (i.e., directed or hands-on) training in conservation biology? Please check all that apply. | Multiple choice | 468 |
| Barriers in conservation physiology | The following list represents potential barriers to the integration of conservation and physiology. Please indicate how often you believe researchers/practitioners face each barrier while attempting to accomplish the goals of Conservation Physiology | Likert scale | 462-467 |
|  | Please indicate how easy you believe each of the following barriers to the integration of conservation and physiology are to overcome. | Likert scale | 457-463 |
|  | Please indicate how often you have **personally** faced each of the following barriers when attempting to integrate physiology and conservation. | Likert scale | 452-457 |
|  | Which of the barriers from the previous question has been the most difficult for you to overcome and why do you believe it has been so difficult? | Open-ended | 315 |
| Success of conservation physiology | The following are the goals that Conservation Physiology aspires to accomplish (as outlined in Cooke et al. 2013 - doi: 10.1093/conphys/cot001). Please indicate how often you believe the field currently accomplishes each goal. | Likert scale | 423-426 |
|  | Has your work combining physiology and conservation been translated into conservation success (i.e., a measurable change in human behaviour, management, or policy)? | Multiple choice | 435 |
|  | Overall, how often do you believe the field of Conservation Physiology influences human behaviour, management, and/or policy? | Likert scale | 431 |
|  | If you believe that Conservation Physiology influences human behaviour, management, and/or policy very often, often, or sometimes, please indicate the primary way(s) you believe it does so. | Open-ended | 165 |
|  | If you believe that Conservation Physiology rarely or never influences human behaviour, management, and/or policy, please indicate the primary reason(s) why you believe this is the case. | Open-ended | 144 |
| Ways forward | Which of the following do you think is most important for a researcher to be successful in Conservation Physiology (i.e., to allow physiological techniques to solve conservation problems)? Please choose one option. | Multiple choice | 419 |
|  | What is most needed to inspire or allow the next generation of biologists (i.e., students currently being trained in biology) to subscribe to Conservation Physiology? | Open-ended | 273 |

**Supplementary Table 2:** Summary of socio-demographic characteristics of survey respondents (n=468).

| **Demographic variable** | **Number of respondents** | **Percentage** |
| --- | --- | --- |
| *Gender* |  |  |
| Female | 165 | 35.6 |
| Male | 290 | 62.5 |
| Non-binary | 2 | 0.4 |
| Preferred not to say | 7 | 1.5 |
| *Age* |  |  |
| 21-30 | 27 | 5.8 |
| 31-40 | 165 | 35.3 |
| 41-50 | 155 | 33.1 |
| 51-60 | 72 | 15.4 |
| 60+ | 45 | 9.6 |
| Preferred not to say | 4 | 0.9 |
| *Education* |  |  |
| College or trades certificate | 2 | 0.4 |
| Undergraduate | 12 | 2.6 |
| Masters | 28 | 6.0 |
| PhD | 410 | 88.0 |
| Habilitation or DSc | 8 | 1.7 |
| Doctor of Veterinary Medicine | 5 | 1.1 |
| Doctor of Medicine | 1 | 0.2 |
| *Sector of employment* |  |  |
| Governmental agency | 44 | 9.4 |
| University/College | 345 | 73.6 |
| Not-for-profit/non-governmental | 20 | 4.3 |
| Private sector | 5 | 1.1 |
| Research institute | 5 | 1.1 |
| Multi-sector | 48 | 10.2 |
| Business proprietor | 1 | 0.2 |
| Unemployed | 1 | 0.2 |
| *Position* |  |  |
| Governmental scientist | 64 | 13.7 |
| Non-governmental scientist | 42 | 9.0 |
| Private sector scientist | 4 | 0.9 |
| Research faculty | 207 | 44.3 |
| Educator/lecturer | 45 | 9.6 |
| Consultant | 3 | 0.6 |
| Manager/administrator | 7 | 1.5 |
| Graduate student or post-doctoral fellow | 82 | 17.6 |
| Technician/research assistant | 9 | 1.9 |
| Veterinarian | 1 | 0.2 |
| Retired/unemployed | 3 | 0.6 |

**Supplementary Table 3:** Demographic characteristics of conservation physiologists and non-conservation physiologists (based on self-identification).

|  | **Non-Conservation Physiologists** | | **Conservation Physiologists** | |
| --- | --- | --- | --- | --- |
|  | **Number of respondents** | **Percentage** | **Number of respondents** | **Percentage** |
| *Position* |  |  |  |  |
| Educator/lecturer | 25 | 9.1 | 17 | 11.6 |
| Graduate student/post-doctoral fellow | 45 | 16.4 | 29 | 19.7 |
| Research faculty | 118 | 42.9 | 58 | 39.5 |
| Private sector scientist | 2 | 0.7 | 1 | 0.7 |
| Other | 17 | 6.2 | 4 | 2.7 |
| Non-governmental scientist | 22 | 8.0 | 10 | 6.8 |
| Governmental scientist | 38 | 13.8 | 20 | 13.6 |
| Consultant | 3 | 1.1 | 0 | 0.0 |
| Technician/research assistant | 1 | 0.4 | 5 | 3.4 |
| Manager/administrator | 4 | 1.5 | 3 | 2.0 |
| *Gender* |  |  |  |  |
| Male | 177 | 64.6 | 93 | 62.8 |
| Female | 91 | 33.2 | 53 | 35.8 |
| Non-binary | 1 | 0.4 | 0 | 0.0 |
| Prefer not to say | 5 | 1.8 | 2 | 1.4 |
| *Age* |  |  |  |  |
| 21-30 | 12 | 4.4 | 10 | 6.8 |
| 31-40 | 98 | 35.6 | 54 | 36.5 |
| 41-50 | 93 | 33.8 | 47 | 31.8 |
| 51-60 | 44 | 16.0 | 22 | 14.9 |
| 60+ | 25 | 9.1 | 14 | 9.5 |
| Prefer not to say | 3 | 1.1 | 1 | 0.7 |

**Supplementary Table 4:** Disciplinary foci of survey respondents (n=542 responses by 379 respondents; i.e., some respondents listed more than one discipline).

| Discipline | Number of responses |
| --- | --- |
| Animal behavior | 4 |
| Animal physiology | 1 |
| Animal science | 1 |
| Applied ecology | 4 |
| Applied population ecology | 1 |
| Aquaculture | 2 |
| Aquatic biology | 1 |
| Aquatic ecology | 2 |
| Avian ecology | 1 |
| Behavior | 7 |
| Behavioral ecology | 17 |
| Behavioral endocrinology | 3 |
| Biochemistry | 2 |
| Biogeochemistry | 2 |
| Biogeography | 2 |
| Biological rhythms | 1 |
| Biology | 1 |
| Botany | 1 |
| Cardiac physiology | 1 |
| Cardiorespiratory physiology | 1 |
| Cell biology | 1 |
| Chemical ecology | 2 |
| Climate change biology | 8 |
| Community ecology | 2 |
| Community physiology | 1 |
| Comparative endocrinology | 1 |
| Comparative immunology | 1 |
| Comparative physiology | 15 |
| Conservation behavior | 1 |
| Conservation biology | 33 |
| Conservation ecology | 9 |
| Conservation endocrinology | 1 |
| Conservation engineering | 1 |
| Conservation genetics | 5 |
| Conservation monitoring | 1 |
| Conservation plant biology | 1 |
| Coral reef science | 1 |
| Cryopreservation | 1 |
| Data science | 1 |
| Dendroecology | 1 |
| Developmental biology | 2 |
| Disease biology | 1 |
| Disease ecology | 4 |
| Ecohydrology | 1 |
| Ecoimmunology | 1 |
| Ecological genomics | 1 |
| Ecological health | 1 |
| Ecology | 71 |
| Ecophysiology | 79 |
| Ecosystem ecology | 1 |
| Ecotoxicology | 2 |
| Endocrinology | 5 |
| Environmental education | 1 |
| Environmental endocrinology | 4 |
| Environmental science | 2 |
| Environmental toxicology | 1 |
| Epidemiology | 2 |
| Epigenetics | 1 |
| Ethno-ornithology | 1 |
| Evolution | 8 |
| Evolutionary biology | 11 |
| Evolutionary ecology | 10 |
| Evolutionary physiology | 9 |
| Fish biology | 2 |
| Fish ecology | 2 |
| Fisheries biology | 4 |
| Fisheries ecology | 1 |
| Fisheries science | 1 |
| Forest conservation biology | 1 |
| Forest ecology | 1 |
| Forest ecophysiology | 1 |
| Forest restoration science | 1 |
| Forest science | 1 |
| Freshwater biology | 1 |
| Freshwater ecology | 2 |
| Functional diversity | 1 |
| Functional morphology | 1 |
| Fundamental science | 1 |
| Genetics | 4 |
| Global change biology | 1 |
| Global change ecology | 1 |
| Herpetology | 4 |
| Immunology | 2 |
| In progress/still determining | 2 |
| Insect conservation biology | 1 |
| Integrative behavior | 1 |
| Integrative biology | 1 |
| Integrative evolutionary ecology | 1 |
| Integrative physiology | 3 |
| Interdisciplinary conservation | 1 |
| Invasion biology | 5 |
| Invasion ecology | 1 |
| Landscape ecology | 1 |
| Macroecology | 2 |
| Macrophysiology | 2 |
| Malacology | 1 |
| Mammalogy | 2 |
| Marine biology | 3 |
| Marine ecology | 6 |
| Marine ecosystem ecology | 1 |
| Marine mammal physiology | 1 |
| Microbial ecology | 1 |
| Microbiology | 1 |
| Modelling | 3 |
| Molecular biology | 4 |
| Molecular ecology | 6 |
| Molecular ecophysiology | 1 |
| Movement ecology | 1 |
| Multivariate statistics | 1 |
| Networking science | 1 |
| Nutritional ecology | 1 |
| Oceanography | 1 |
| Ornithology | 1 |
| Physiology | 13 |
| Plant biology | 2 |
| Plant conservation biology | 2 |
| Plant ecology | 6 |
| Plant Ecophysiology | 12 |
| Plant physiology | 5 |
| Plant-climate interactions | 1 |
| Population biology | 1 |
| Population ecology | 8 |
| Predictive ecology | 1 |
| Primatology | 1 |
| Quantitative ecology | 1 |
| Reproductive biology | 3 |
| Reproductive physiology | 2 |
| Restoration ecology | 4 |
| Restoration science | 1 |
| Seagrass ecology | 1 |
| Seed science | 1 |
| Sociologial ecology | 1 |
| Statistical science | 1 |
| Systems biology | 1 |
| Theoretical ecology | 1 |
| Thermal biology | 2 |
| Toxicology | 3 |
| Tree physiology | 1 |
| Veterinary science | 2 |
| Veterminary medicine | 1 |
| Wildlife biology | 3 |
| Wildlife disease | 1 |
| Wildlife ecology | 2 |
| Wildlife endocrinology | 1 |
| Wildlife management | 1 |
| Wildlife medicine | 2 |
| Wildlife science | 2 |

**Supplementary Table 5:** Remaining responses provided for why conservation physiology 'rarely' or 'never' successful at changing human behaviour, management, or policy.

| **Response (thematized)** | **Count** | **Percent** |
| --- | --- | --- |
| Public is not swayed by science | 2 | 1.3 |
| Changing policy requires more than good science | 1 | 0.7 |
| Conservation in any form does not influence human behaviour as much as we hope | 1 | 0.7 |
| Current political climate (United States) | 1 | 0.7 |
| Hard to shape actionable message to decision-makers | 1 | 0.7 |
| Inability of scientists to identify which risks/stressors are most important | 1 | 0.7 |
| Lack of connection between conservation and physiology | 1 | 0.7 |
| Managers have trouble connecting physiology and conservation | 1 | 0.7 |
| Physiological data are not available for most major conservation problems | 1 | 0.7 |
| Public is unaware that they need to change | 1 | 0.7 |
| Public not thinking about science | 1 | 0.7 |
| Too many pressures and influences | 1 | 0.7 |
| Unstable funding | 1 | 0.7 |

**Supplementary Figure 1:** Taxonomic focus of survey respondents. In total, 468 respondents reported 695 topics of focus (i.e., some individuals work on multiple taxa).

**Supplementary Figure 2:** Frequency with which participants take a A) physiological and B) conservation approach in their current work, partitioned by self-identification as a conservation physiologist.

**Conservation Physiologists** **Non-Conservation Physiologists**
